# Pulsed Field Ablation for the Treatment of Ventricular Arrhythmias Using a Focal, Contact-Force Sensing Catheter: A Single-Center Case Series and Review

**DOI:** 10.3390/jcdd13020059

**Published:** 2026-01-23

**Authors:** Cristian Martignani, Giulia Massaro, Alberto Spadotto, Maria Carelli, Lorenzo Bartoli, Alessandro Carecci, Andrea Angeletti, Matteo Ziacchi, Mauro Biffi, Matteo Bertini

**Affiliations:** 1 Institute of Cardiology, IRCCS Azienda Ospedaliero-Universitaria di Bologna, Via Massarenti 9, 40138 Bologna, Italy; 2 Department of Medical and Surgical Sciences, Institute of Cardiology, University of Bologna, Via Massarenti 9, 40138 Bologna, Italy; 3 Cardiology Unit, Department of Translational Medicine, Sant’Anna University Hospital, University of Ferrara, 44124 Ferrara, Italy

**Keywords:** pulsed field ablation, ventricular tachycardia, premature ventricular complexes, catheter ablation, Centauri system, irreversible electroporation, ventricular arrhythmias

## Abstract

Background: Catheter ablation is a validated treatment for ventricular arrhythmias (VA), but conventional radiofrequency (RF) energy may cause collateral injury due to non-selective thermal damage. Pulsed Field Ablation (PFA), a non-thermal modality based on irreversible electroporation, offers myocardial tissue selectivity and enhanced safety. While PFA is widely adopted for atrial arrhythmias’ ablation, its application in the ventricles remains an evolving frontier. Methods: We report a single-center experience using the Centauri PFA system integrated with a focal, contact-force sensing irrigated catheter (Tacticath™ SE, Abbott Laboratories, St. Paul, MN, USA) in four consecutive patients with drug-refractory VA. Two patients presented with frequent premature ventricular complexes (PVC) arising from the right and left ventricular outflow tract, respectively, while two had ischemic cardiomyopathy with recurrent scar-related ventricular tachycardia (VT). All procedures were guided by high-density mapping using the EnSite X system (Abbott Laboratories, St. Paul, MN, USA). Procedural safety, acute efficacy, and early follow-up outcomes were assessed. Results: All ablations achieved acute procedural success without complications. In both PVC cases, PFA led to immediate and complete suppression of ectopy, with a ≥95% reduction in arrhythmic burden at 12- and 9-months follow-up, respectively. In the VT cases, the arrhythmogenic substrate was effectively modified, rendering the clinical VT non-inducible. ICD interrogation during a 9-month follow-up showed complete absence of recurrent sustained VT. No coronary spasm, atrioventricular block, pericardial effusion, or other adverse events occurred. Conclusions: In this initial experience, focal PFA using a contact-force sensing catheter appeared feasible and effective for both focal and scar-related VA. This system provides an intuitive workflow similar to RF ablation. While our data suggest a favourable safety profile, larger studies are required to definitively confirm safety margins near critical structures.

## 1. Introduction

Ventricular arrhythmias (VA), encompassing a spectrum from frequent premature ventricular complexes (PVC) to life-threatening ventricular tachycardia (VT), represent a significant clinical challenge. They are major contributors to cardiovascular morbidity and mortality worldwide. Specifically, a high burden of PVCs can lead to tachycardia-induced cardiomyopathy and debilitating symptoms, whereas VT, particularly in the context of ischemic or non-ischemic structural heart disease, remains a leading cause of sudden cardiac death (SCD) [1]. While antiarrhythmic drugs (AADs) are often the first line of therapy, they are frequently limited by modest efficacy and significant side effects. Consequently, catheter ablation became the mainstay of therapy for patients with drug-refractory or symptomatic VA [1].

Classically, catheter ablation is performed using thermal energy sources, most notably radiofrequency (RF) or cryothermal energy. RF ablation creates lesions via resistive heating, resulting in coagulative necrosis of the target myocardium. Although this modality is highly effective, it possesses inherent limitations driven by the non-selective nature of thermal injury. The spread of heat into surrounding tissues can cause collateral damage to critical structures such as the coronary arteries, the His–Purkinje conduction system, the phrenic nerve, and the oesophagus. Furthermore, the physics of RF delivery in the thick ventricular myocardium can lead to complications such as steam pops (intramural explosions due to tissue overheating), thrombus formation on the catheter tip, and charring, which may result in embolic stroke or cardiac tamponade [2].

In recent years, Pulsed Field Ablation (PFA) has emerged as a disruptive technology in cardiac electrophysiology. PFA relies on the biophysical principle of irreversible electroporation (IRE). Instead of thermal energy, PFA delivers ultra-short, high-voltage electrical pulse trains that create a high-intensity electric field. This field destabilizes cell membranes by forming permanent nanopores, leading to disruption of homeostasis and subsequent cell death via apoptosis or necrosis [3]. A critical advantage of PFA is its tissue specificity: myocardial cells have a lower threshold for electroporation compared to surrounding connective tissue, vascular endothelium, and nerves [4,5]. This differential sensitivity theoretically allows for the transmural ablation of arrhythmogenic tissue while sparing adjacent coronary arteries and conduction pathways.

While PFA has been rapidly adopted as a standard of care for atrial fibrillation (AF) ablation due to its speed and safety profile, its application in the ventricular chambers is still considered an evolving frontier. The ventricles present unique challenges, including a significantly thicker myocardium compared to the atria, complex three-dimensional geometry, and the immediate proximity of major coronary vessels. Preclinical studies and early human trials, such as the VCAS (Ventricular Catheter Ablation with Pulsed Field) trial [6], began to demonstrate that PFA can create deep, transmural, and durable lesions in the ventricular myocardium.

In this study, we present our early single-center experience using the Centauri PFA system. Unlike single-shot PFA catheters designed for pulmonary vein isolation, this system integrates with a standard focal, contact-force sensing RF catheter. This allows for precise, point-by-point ablation of complex ventricular substrates. We describe the methodology and outcomes of four consecutive cases of both focal and scar-related VA, aiming to provide detailed insight into the procedural workflow, safety profile, and mid-term efficacy of this novel therapeutic approach.

## 2. Materials and Methods

### 2.1. Patient Population and Study Design

This is a single-center, observational case series involving four consecutive patients who underwent PFA for symptomatic, drug-refractory VA at the Cardiology Unit of IRCCS Azienda Ospedaliero-Universitaria di Bologna, Policlinico S. Orsola-Malpighi.

Patients were considered eligible for inclusion if they met the following criteria:

1. Documented frequent PVCs (>10,000 beats/24 h) associated with severe symptoms or decline in left ventricular (LV) function (PVC-induced cardiomyopathy).
2. Structural heart disease with recurrent sustained VT or arrhythmic storms occurring despite optimal medical therapy, including antiarrhythmic drugs (amiodarone, beta-blockers, or sotalol).
3. Refractoriness or intolerance to at least one antiarrhythmic drug.

All procedures were conducted in accordance with the Declaration of Helsinki. Written informed consent was obtained from all patients prior to the procedure, covering both the ablation procedure and the collection of clinical data for research purposes, following approval by the local Institutional Review Board. Baseline clinical evaluation included a comprehensive 12-lead ECG analysis to localize the arrhythmia exit site, transthoracic echocardiography to assess structural heart disease and ejection fraction, and 24-h Holter monitoring to quantify arrhythmic burden.

### 2.2. Pre-Procedural Preparation

Procedures were performed under general anaesthesia to ensure patient immobility and stable conditions, particularly important during the delivery of high-voltage PFA applications which can induce skeletal muscle contraction. Invasive hemodynamic monitoring (arterial line) and surface ECG monitoring were maintained throughout. Defibrillator pads were positioned anteriorly and posteriorly.

### 2.3. Electrophysiological Mapping and Setup

Vascular accesses were obtained via femoral veins for right ventricular procedures and transseptal puncture. For left ventricular access, a retrograde aortic approach via femoral artery was employed when necessary, depending on the arrhythmia origin. Systemic heparinization was administered to maintain an activated clotting time (ACT) > 300 s for left-sided procedures.

A high-density 3D electroanatomic map was created using the EnSite X impedance-magnetic field mapping system (Abbott Laboratories, St. Paul, MN, USA). The mapping strategy differed based on the arrhythmia type:

- For PVC cases: Activation mapping was performed during spontaneous ectopy or isoproterenol-induced ectopy. The focus was identified by searching for the local electrogram preceding the surface QRS onset by the greatest interval (typically >20–30 ms) with a QS morphology on the unipolar signal. Pace mapping was utilized to confirm a ≥95% match between the paced QRS morphology and the clinical PVC 12-lead ECG.
- For VT cases: High-density substrate mapping was performed during sinus rhythm using a multipolar HD Grid catheter (Abbott Laboratories, St. Paul, MN, USA). Bipolar voltage settings were standardized, with <0.5 mV defining dense scar and 0.5–1.5 mV defining border zones/healthy tissue. Areas of slow conduction were identified by the presence of late potentials (LP) or fractionated electrograms. Programmed ventricular stimulation (up to 3 extra-stimuli) was used to induce the clinical VT. If the VT was hemodynamically tolerated, entrainment mapping was performed to identify the critical isthmus; otherwise, a substrate-based modification strategy was employed.

### 2.4. PFA System and Protocol

The ablation setup utilized the Centauri PFA System (CardioFocus Inc., Marlborough, MA, USA). This proprietary system acts as a generator and a switching unit that allows the use of standard commercially available focal ablation catheters. In this series, the generator was connected to a Tacticath™ SE contact-force sensing, irrigated-tip catheter (Abbott Laboratories, St. Paul, MN, USA). This integration preserves the “RF-like” workflow, maintaining real-time visualization of the catheter tip on the mapping system, impedance monitoring, and crucially contact-force feedback [7].

The Centauri system delivers a proprietary biphasic, high-voltage pulse train. The energy delivery is synchronized with the cardiac cycle (triggered on the R-wave) to avoid the vulnerable period of repolarization and minimize the risk of inducing ventricular fibrillation.

- Targeting: PFA was delivered at each target site only when stable catheter contact was confirmed (Contact Force > 10 g).
- Delivery Parameters: The generator current was set to 25 A. Each energy application consisted of 10 trains composed of 3 pulses. Typically, 1 application per site was delivered until the complete elimination of the local electrogram and impedance drop were observed, consistent with preclinical findings on focal PFA lesion formation [8].
- Irrigation: The catheter irrigation flow rate was maintained at 4 mL/min during energy delivery to prevent any potential heating of the electrode, although the primary mechanism is non-thermal.

In the first case [right ventricular outflow tract (RVOT) PVC], intravenous nitrates were infused prophylactically to prevent coronary spasm, reflecting early caution with the technology. In subsequent cases, based on accumulating safety data, nitrates were omitted without incident.

### 2.5. Procedural Endpoints and Follow-Up

The procedural endpoints were defined as follows:

- PVC cases: Complete abolition of spontaneous ectopy for a waiting period of at least 30 min post-ablation, inclusive of isoproterenol challenge, and non-inducibility on programmed stimulation.
- VT cases: Termination of VT during PFA delivery (if applicable) and, primarily, the non-inducibility of any sustained VT at the end of the procedure using an aggressive stimulation protocol. Complete elimination of local abnormal ventricular activities and LP in the target substrate was also required.

Post-procedure, all patients underwent transthoracic echocardiography to rule out pericardial effusion. Patients were monitored on telemetry for 24–48 h. Follow-up consisted of clinical visits, 12-lead ECGs, and 24-h Holter monitoring or ICD interrogation at 3, 6, 9, and 12 months (where applicable) to assess long-term efficacy and safety.

## 3. Results

All four consecutive procedures achieved 100% acute technical success. No procedural complications, including steam pops, charring, tamponade, or vascular access issues were recorded. Detailed patient and procedural data are provided in Table 1.

*Case 1—RVOT PVC*

**Figure 1:**
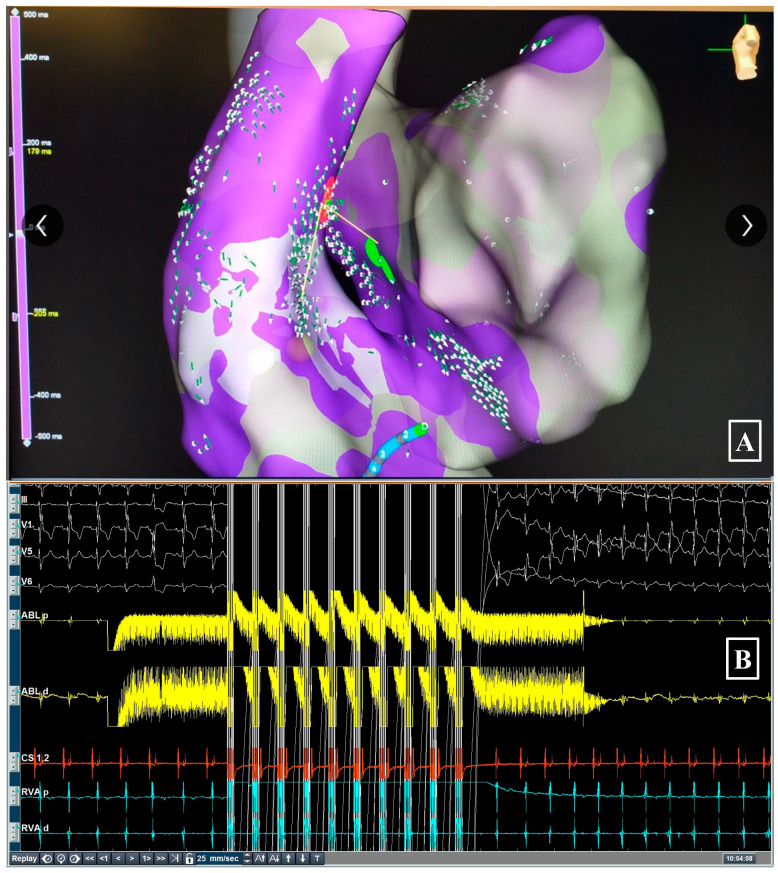
(**Panel A**) 3D activation map of RVOT PVC case showing earliest activation (red zone) near His bundle (green zone). (**Panel B**) ECG and local electrograms before and after PFA, showing elimination of PVC.

A 58-year-old male presented with palpitations and mild left ventricular dysfunction (LVEF 45%), suspected to be PVC-induced cardiomyopathy. 24-h Holter monitoring revealed a 25% burden of monomorphic PVCs. Beta-blocker therapy failed to reduce PVC burden. Electroanatomic activation mapping localized the earliest activation site to the septal aspect of the RVOT base, approximately 15 mm from the His bundle region (Figure 1). Although PFA is tissue-selective, the His bundle is composed of cardiomyocytes and is therefore susceptible to irreversible electroporation. Consequently, despite the anatomical safety margin, energy was delivered with caution. A single focal PFA application resulted in the immediate cessation of PVCs. Post-ablation testing showed intact AV node conduction with no change in PR interval. No ST-segment elevation was observed. At the 12-month follow-up, Holter monitoring demonstrated a 98% reduction in PVC burden (from 26,000 to 520/24 h), and echocardiography showed normalization of LVEF to 55%. The patient remains asymptomatic without antiarrhythmic medication.

*Case 2—Left Ventricular Outflow Tract (LVOT) PVC*

A 62-year-old female with preserved LVEF but debilitating symptoms presented with an 18% PVC burden refractory to medical therapy. The 12-lead ECG suggested an origin from the left coronary cusp (LCC). The aorta was accessed retrograde. Activation mapping confirmed the earliest site within the LCC. While RF ablation at the base of the cusp is generally safe, thermal injury to coronary ostia remains a concern in complex anatomies, of consequence PFA was selected as an alternative modality. Energy was delivered via the Tacticath catheter under stable contact. The application resulted in immediate elimination of the ectopy. Coronary angiography was not performed pre- or post-ablation as no ECG signs of ischemia were noted; however, we acknowledge that subclinical spasm cannot be ruled out. The patient was discharged the following day. At 9 months, PVC burden was reduced by 95% (<1000/24 h), and the patient reported complete resolution of palpitations.

*Case 3—Ischemic VT (Infero-postero-lateral LV scar)*

A 68-year-old male with a history of inferior myocardial infarction and ischemic cardiopathy (LVEF 35%) presented with an electrical storm. He had a secondary prevention dual-chamber ICD and was on amiodarone. Substrate mapping using the HD Grid catheter identified a dense scar in the infero-postero-lateral wall extending to the base. Extensive LP were recorded in the border zone. Clinical VT was induced and tolerated, allowing for entrainment mapping which localized the critical isthmus within the scar channel. PFA was delivered to transect the isthmus and homogenize the substrate containing LP. The VT terminated during the first application. Subsequent remodelling of the scar area resulted in non-inducibility of any VT. Over a 9-month follow-up, ICD interrogation confirmed no episodes of sustained VT and no shock therapies.

*Case 4—Ischemic VT (Antero-septal LV scar)*

**Figure 2:**
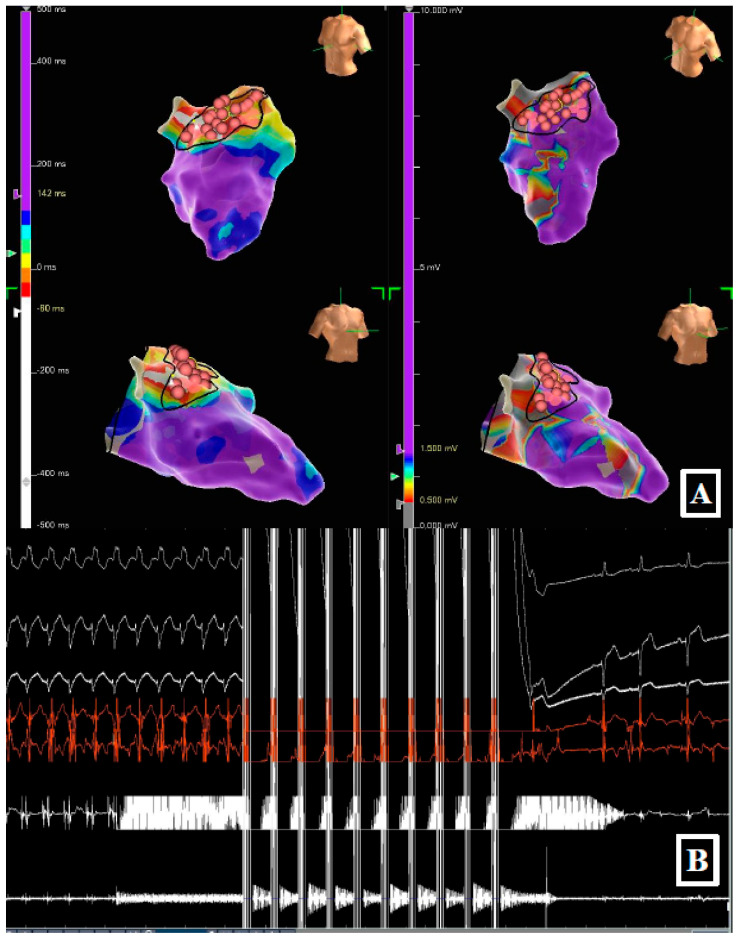
(**Panel A**) High-density activation map (**left**) and bipolar voltage map (**right**) of the left ventricle, characterizing the antero-septal substrate. (**Panel B**) Real-time recording during ablation. Selected surface leads (highlighting the RBBB morphology in V1) and intracardiac electrograms demonstrate the delivery of the PFA train (white artifact) followed by the immediate termination of the ventricular tachycardia.

A 71-year-old male with ischemic cardiopathy (LVEF 30%), prior coronary bypass grafting and previous ventricular septal defect repair with a Dacron patch, was admitted for recurrent monomorphic VT treated by multiple ICD shocks. The clinical VT (RBBB morphology) was inducible at baseline (Figure 2). Mapping revealed a complex heterogeneous substrate with areas of slow conduction in the antero-septal LV. Due to the thickness of the septum and the presence of surgical patch material, achieving transmurality with RF was considered challenging. Targeted PFA applications were delivered to eliminate fragmented electrograms and define a line of block. The procedure successfully rendered the patient non-inducible. At 9 months, the patient remained free from VT recurrence and arrhythmic storm.

## 4. Discussion

This single-center case series and review highlight the safety, feasibility, and efficacy of focal PFA for the treatment of VA. By utilizing a hybrid system that pairs a PFA generator with a standard contact-force sensing irrigated catheter, we successfully treated a spectrum of pathologies ranging from focal outflow tract PVC to complex scar-related VT in ischemic cardiopathy.

### 4.1. Mechanism of Safety and Efficacy

The fundamental advantage of PFA lies in its non-thermal mechanism. PFA induces cell death by creating nanopores in the sarcolemma, a process known as IRE [3]. Unlike RF, which relies on conductive heating, PFA fields are essentially transparent to the extracellular matrix. This results in the preservation of the structural integrity of the tissue, sparing the collagen architecture, nerves, and blood vessels.

Our clinical observations support the preclinical data regarding tissue selectivity [4,5]. In Case 1, ablation was performed near the His bundle without compromising AV conduction. In Case 2, ablation within the aortic cusp, a high-risk site for thermal injury to coronary ostia, was performed without coronary spasm or stenosis. These findings are consistent with the VCAS trial [6] and recent reports by Padisak et al. [9], which suggest that PFA may allow for safer ablation of targets that were previously considered “no-fly zones” for thermal energy. The absence of ST-segment elevation during energy delivery in our series further corroborates the lack of collateral thermal injury to the coronary microvasculature.

### 4.2. Workflow Integration and Catheter Technology

A significant barrier to the adoption of new technologies is often the learning curve associated with novel catheter designs. Most current PFA systems for AF utilize “single-shot” catheters (penta-spline or lattice tips) which are bulky and ill-suited for the precise point-by-point mapping required in ventricular ablation. The Centauri system addresses this by enabling PFA delivery through a standard focal catheter (Tacticath, Abbott). This allows the operator to utilize familiar manoeuvres, contact-force feedback, and high-density mapping systems (EnSite X, Abbott) without modification [10].

This “RF-like” workflow is crucial for ventricular procedures where stable catheter contact is a major determinant of lesion depth. Nakagawa et al. [2] and recent studies [11] emphasized that adequate contact force is as critical for PFA as it is for RF to ensure transmurality in the thick ventricular wall. The contact-force feedback in our series ensured that energy was only delivered when the catheter was adequately coupled to the tissue (>10 g), likely contributing to our 100% acute success rate.

### 4.3. Comparison with Current Literature

Our results align with and expand upon emerging literature. Recently, Peichl et al. [12] reported the results of a large multicenter study on focal PFA for VA, demonstrating high acute success rates and a favourable safety profile. Similarly, the VCAS trial reported 96% acute success in scar-related VT [6]. However, that trial utilized a dedicated catheter system. Our study confirms that similar efficacy can be achieved with a generic focal catheter interface. Furthermore, Verhaeghe et al. [13] recently reported PFA as a successful bailout strategy after failed RF ablation. Our series suggests PFA is also viable as a first-line therapy. The durability of the lesions is evidenced by the significant reduction in PVC burden and freedom from VT at 9–12 months follow-up, comparable to historical RF data but with a potentially superior safety profile.

### 4.4. Safety Considerations

The fundamental advantage of PFA lies in its non-thermal mechanism. However, safety claims must be interpreted with caution given the small sample size.

Regarding conduction system safety, our experience in Case 1 suggests that PFA can be delivered in the para-Hisian region. However, it is crucial to emphasize that the His bundle is not immune to PFA. The safety we observed was likely due to the anatomical distance (15 mm) rather than intrinsic resistance of the conduction axis.

Regarding coronary safety (Case 2), PFA theoretically spares the collagen-rich architecture of blood vessels. However, PFA-induced coronary vasospasm is an emerging concern described in the recent literature. In our series, we did not perform systematic angiography, which limits our ability to exclude subclinical vascular effects. Future protocols should strongly consider angiographic assessment when ablating near epicardial vessels.

### 4.5. Future Directions

The application of PFA in the ventricles is in its infancy. Future research should focus on optimizing dosing protocols (voltage, pulse width, and number of trains) specifically for the variable thickness of the ventricular myocardium. While our series utilized a focal approach, the development of large-footprint PFA catheters capable of rapid substrate homogenization could further reduce procedure times for ischemic VT. Additionally, the integration of PFA with real-time MRI or advanced impedance mapping could provide immediate confirmation of lesion transmurality, a capability currently lacking in standard practice. Large-scale, multicenter randomized controlled trials comparing PFA directly to RF are necessary to definitively establish long-term non-inferiority or superiority.

## 5. Limitations

This study is subject to the inherent limitations of a small, single-center case series (4 patients)**.** This sample size precludes definitive conclusions regarding rare adverse events and general safety. Follow-up was limited to 9–12 months, and we did not utilize post-procedural cardiac MRI to quantify lesion size. Finally, as PFA waveforms and catheter geometries vary significantly between manufacturers (e.g., Centauri vs. Farawave), safety and efficacy data from this study are specific to the system and settings used (25 A, focal catheter) and cannot be automatically extrapolated to other PFA platforms.

## 6. Clinical Implications

Our findings underscore the transformative potential of PFA in the management of VA. By integrating a focal contact-force catheter with a non-thermal energy source, PFA enables precise tissue-selective lesion creation while minimizing the risk of collateral damage. Clinically, this approach may redefine safety thresholds for ablation near coronary arteries, conduction tissue, or prosthetic material. The reproducibility of outcomes and compatibility with existing mapping platforms suggest that PFA could be seamlessly integrated into routine clinical workflows. As technology evolves, PFA could become the preferred modality for VA, particularly in patients with complex anatomy or prior failed RF ablations, marking a paradigm shift toward safer and more predictable substrate modification.

## 7. Conclusions

In this preliminary experience, PFA using a focal, contact-force sensing catheter appeared feasible, safe, and effective for treating both focal PVC and scar-related VT. This technology combines procedural familiarity with the unique benefits of non-thermal ablation. While our initial results are promising, larger prospective trials are necessary to standardize dosing protocols and confirm long-term safety, particularly regarding the conduction system and coronary vasculature.

## Figures and Tables

**Table 1:** Patient and Procedural Characteristics.

Characteristic	Case 1	Case 2	Case 3	Case 4
Age/Sex	58/M	62/F	68/M	71/M
Arrhythmia Type	PVC	PVC	Ischemic VT	Ischemic VT
LVEF (%)	45	50	35	30
Target Site	RVOT base	LVOT (aortic cusp)	Infero-postero-lateral LV	Antero-septal LV
Mapping System	EnSite X	EnSite X	EnSite X + HD Grid	EnSite X + HD Grid
Procedure Duration (min)	90	110	180	170
Acute Success	Yes	Yes	Yes	Yes
Complications	None	None	None	None
Follow-up Duration	12 mo	9 mo	9 mo	9 mo

F, female; HD, high density; LV, left ventricle; LVEF, left ventricular ejection fraction; LVOT, left ventricular outflow tract; M, male; mo, months; PVC, premature ventricular complex; RVOT, right ventricular outflow tract; VT, ventricular tachycardia.

## Data Availability

The original contributions presented in this study are included in the article. Further inquiries can be directed to the corresponding author.
